# Retinal Pigment Epithelium Sequelae Caused by Blunt Ocular Trauma: Incidence, Visual Outcome, and Associated Factors

**DOI:** 10.1038/s41598-017-14659-4

**Published:** 2017-10-27

**Authors:** Seong Joon Ahn, Se Joon Woo, Kyu Hyung Park, Byung Ro Lee

**Affiliations:** 1 Department of Ophthalmology, Hanyang University Hospital, Seoul, South Korea; 2 Department of Ophthalmology, Armed Forces Capital Hospital, Seongnam, South Korea; 3 Department of Ophthalmology, Seoul National University Bundang Hospital, Seongnam, South Korea

## Abstract

Vision loss can occur in eyes with blunt ocular trauma, but the causes have not been elucidated fully. We encountered cases of retinal pigment epithelium (RPE) sequelae following blunt ocular trauma associated with permanent vision loss in our cohort of patients with blunt ocular trauma. In this multicentre retrospective cohort study on 129 patients with retinal abnormalities caused by acute blunt ocular trauma, we investigated the incidence of RPE sequelae and evaluated associated factors and visual outcomes. RPE sequelae, which typically presented as hyperpigmentation within well-demarcated hypopigmented lesions, occurred in 29 (22.5%) patients within 1 month of trauma. Optical coherence tomography (OCT) revealed complete photoreceptor loss over the abnormal RPE. Final visual outcomes were significantly different between eyes with and without RPE sequelae. Logistic regression analysis revealed a significant association between the presence of subretinal fluid and RPE sequelae. In conclusion, RPE sequelae occurred in approximately 20% of patients with blunt ocular trauma and was associated with permanent photoreceptor defects and visual loss. Clinical evaluation using OCT may help predict RPE sequelae and visual outcomes in eyes with blunt trauma.

## Introduction

Ocular injury is quite common. Approximately two million people in the United States are annually estimated to experience an eye injury requiring treatment^1^, and the rate of emergency-department-treated eye injury has been estimated to be 3.15 per 1000 population^2^. Traumatic eye injuries are one of the most common causes of monocular blindness in the United States, with more than 40,000 new cases of impaired vision reported every year^3^. As injury rates are highest among people in their 20 s and 30 s, the socioeconomic impact and lost productivity in the workplace are significant; eye injuries account for more than 140,000 disabling work injuries every year, with direct and indirect costs estimated at over $4 billion^4^.

Several types of retinal changes can occur after blunt ocular trauma, including commotio retinae, choroidal rupture, subretinal haemorrhage, and macular hole. Previous studies have reported on the visual outcomes of eyes with acute blunt trauma^5,6^. For example, Blanch *et al*. reported on the visual outcomes of eyes with blunt ocular trauma, showing worse visual outcomes in eyes with macular commotio retinae than in those with extramacular commotio retinae^5^. In eyes with blunt ocular trauma, the retinal pigment epithelium (RPE) can also be affected^7^. Friberg *et al*. reported the finding of retinal cloudiness with a cream-coloured discolouration of the RPE, described as ‘RPE oedema’, which subsequently resulted in depigmentation and pigment clumping^7^. Similar findings have also been called RPE contusion^8^. While the effect of damage to the photoreceptor layers on visual outcomes has been relatively well documented^6^, the incidence and clinical significance of RPE sequelae caused by blunt ocular trauma have not been addressed.

Here, we investigated the incidence of RPE sequelae among patients with retinal abnormalities caused by blunt trauma and evaluated the visual outcome of their eyes. To identify predictive factors for the sequelae, we evaluated the baseline clinical factors associated with RPE sequelae. We further assessed morphologic changes in the retina over time by using spectral-domain optical coherence tomography (SD-OCT) to provide structural bases for its clinical significance and explored findings associated with the development of RPE sequelae.

## Results

### Patient and ocular characteristics

The demographic data of patients in this study and the characteristics of eyes with acute blunt trauma are shown in Table 1. The mean age of the patients was 24.6 ± 10.1 years (range: 12–68 years). Of the 129 patients, 122 (94.6%) were male. The mean follow-up period was 4.6 ± 6.6 months (range: 2–60 months). The time after injury to presentation was 21.4 ± 27.5 hours on average (range: 1−168 hours) and the median time was 12 hours (interquartile range, 4–24). Most of the blunt injuries resulted from sports events (81 of 129, 62.8%); the most common cause of trauma was the soccer ball (59 of 129, 45.7%; Supplementary Table). Anterior segment findings included hyphaema (64 of 129, 49.6%) and traumatic iritis (30 of 129, 23.3%).

**Table 1:** Demographic and clinical characteristics of patients with and without retinal pigment epithelium (RPE) sequelae. Data are denoted as number of patients (%) or mean ± standard deviation.

Characteristics	Total (n = 129)	With RPE sequelae (n = 29)	Without RPE sequelae (n = 100)	*P*
Age, yr	24.6 ± 10.1 (range: 12–68)	24.4 ± 10.3	24.6 ± 10.0	0.918
Sex, male:female (%)	7:122 (5.4:94.6)	1:28 (3.4:96.6)	6:94 (6:94)	1.000
Refractive errors, D	−1.63 ± 1.70 (range: −5.75– + 1.25)	−1.07 ± 1.84	−1.80 ± 0.87	0.071
Best-corrected visual acuities at baseline, logMAR	0.50 ± 0.66 (range: LP–20/20)	1.05 ± 0.71	0.34 ± 0.55	<0.001
Orbital wall fracture, No. of patients/No. of CT performed (%)	22/120 (18.3)	8/27 (29.6)	14/93 (15.1)	0.085
Hyphaema	64 (49.6)	15 (51.7)	49 (49)	0.796
Subretinal fluid on OCT at baseline, No. of patients/No. of OCT performed (%)	14/119 (11.8)	11/26 (42.3)	3/93 (3.2)	<0.001
Choroidal rupture (%)	10 (7.8)	7 (24.1)	3 (3)	0.001
Macular commotio retinae grade on OCT, 0:1:2:3:4 (%)	11:64:11:19:14 (9.2:53.8:9.2:16.0:11.8)	0:0:0:15:11 (57.7:42.3)	11:64:11:4:3 (11.8:68.8:11.8:4.3:3.2)	<0.001

CT = computed tomography; LP = light perception; No. = number; OCT = optical coherence tomography.

At baseline, commotio retinae was the most common retinal abnormality in the included patients (121 of 129, 93.8%). Macular and extramacular commotio retinae were noted in 98 (76.0%) and 46 (35.7%) patients, respectively. Both types of commotio retinae were observed in 23 (17.8%) eyes. Among the 119 patients in whom OCT was performed, 64 (53.8% of eyes that underwent OCT) had Grade 1 commotio retinae^6^; 11 (9.2%) had Grade 2; 19 (16.0%) had Grade 3; and 14 (11.8%) had Grade 4. Subretinal fluid (SRF) was noted in 14 (11.8%) patients. Choroidal rupture was noted in 10 of 129 (7.8%) patients by fundus photography or FA.

**Figure 1:**
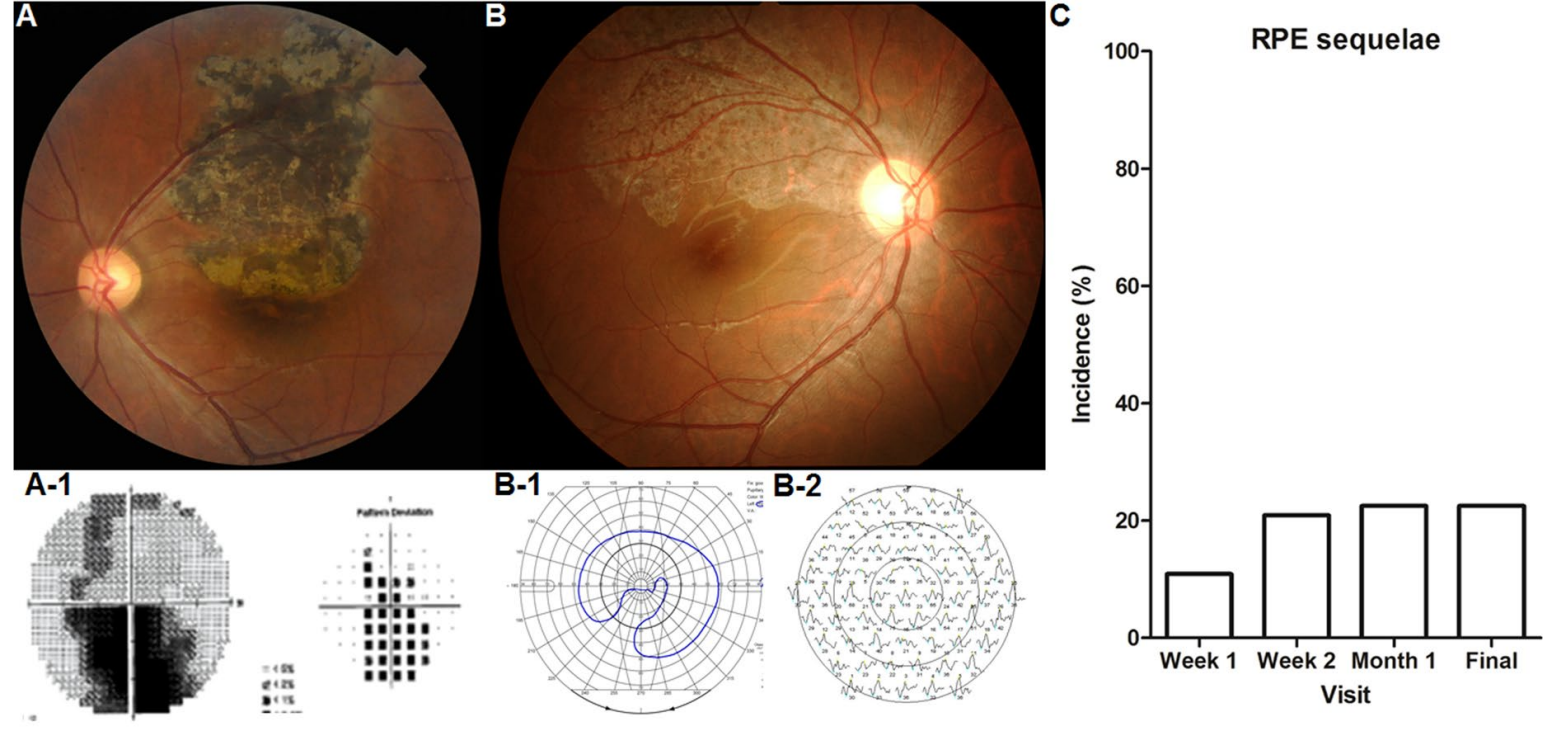
Examples of fundus photographs and visual field examinations in patients with blunt trauma showing retinal pigment epithelium (RPE) sequelae. (A-1) A 35-year-old man with a soccer-ball-induced ocular trauma showing a well-demarcated hypopigmented or hyperpigmented lesion in the superior retina to the fovea. (A-2) Humphrey 30-2 visual field examination shows a corresponding inferior visual field defect. (B-1) A 14-year-old boy with a soccer-ball-induced ocular trauma showing RPE sequelae, resembling a comet shape radiating from the optic disc. Goldmann visual field examination (B-2) and multifocal electroretinogram (B-3) show an inferior visual field defect and decreased response in the superior pericentral area, respectively. (**C**) Incidence of RPE sequelae over time indicates that the sequelae initially occur during the first month after blunt ocular trauma.

**Figure 2:**
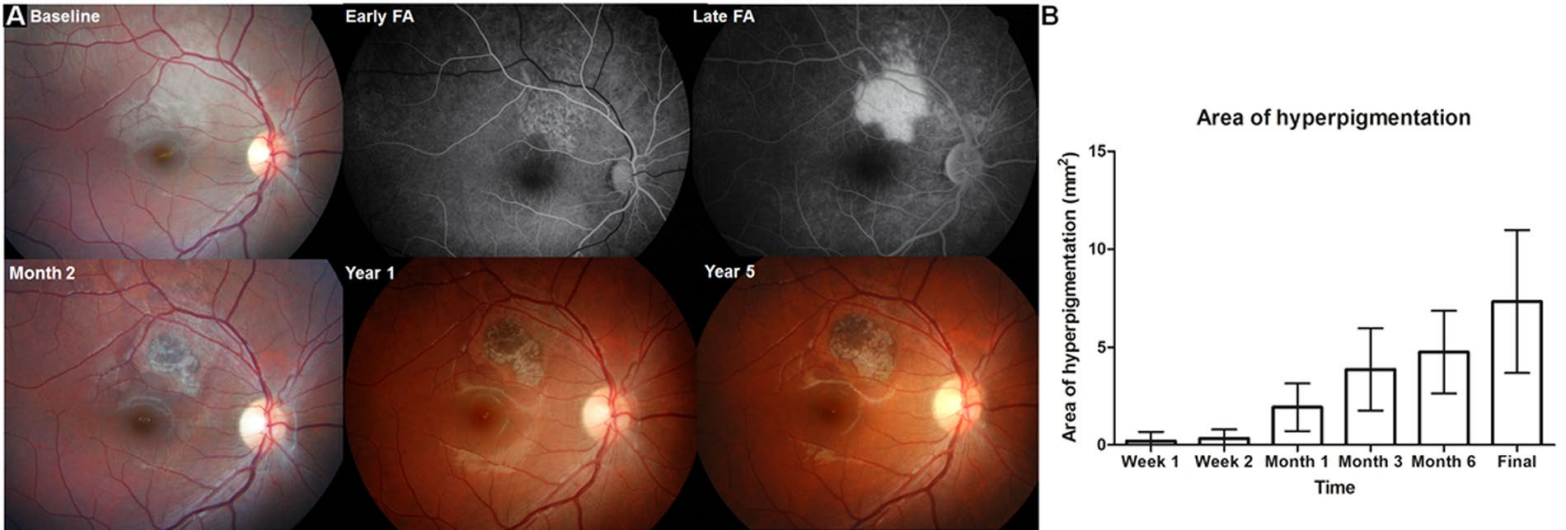
Baseline and follow-up fundus photographs demonstrating the development and progression of retinal pigment epithelium sequelae and their association with baseline fluorescein angiography (FA) findings. (**A**) At baseline (top row), a cream-coloured change of the fundus was noted in the superior retina. FA reveals a localised hyperfluorescent lesion with fine hypofluorescent clumpings at the early phase and leakage over the lesion at the late phase. Subsequent fundus photographs (bottom) showing amorphous hyperpigmentation within a well-demarcated hypopigmented lesion, which corresponds to the area of leakage on FA. The hyperpigmentation gradually increased in size over 5 years. (**B**) The mean area of hyperpigmentation in eyes showing pigmentation gradually increases over time. Error bars indicate 95% confidence intervals.

**Figure 3:**
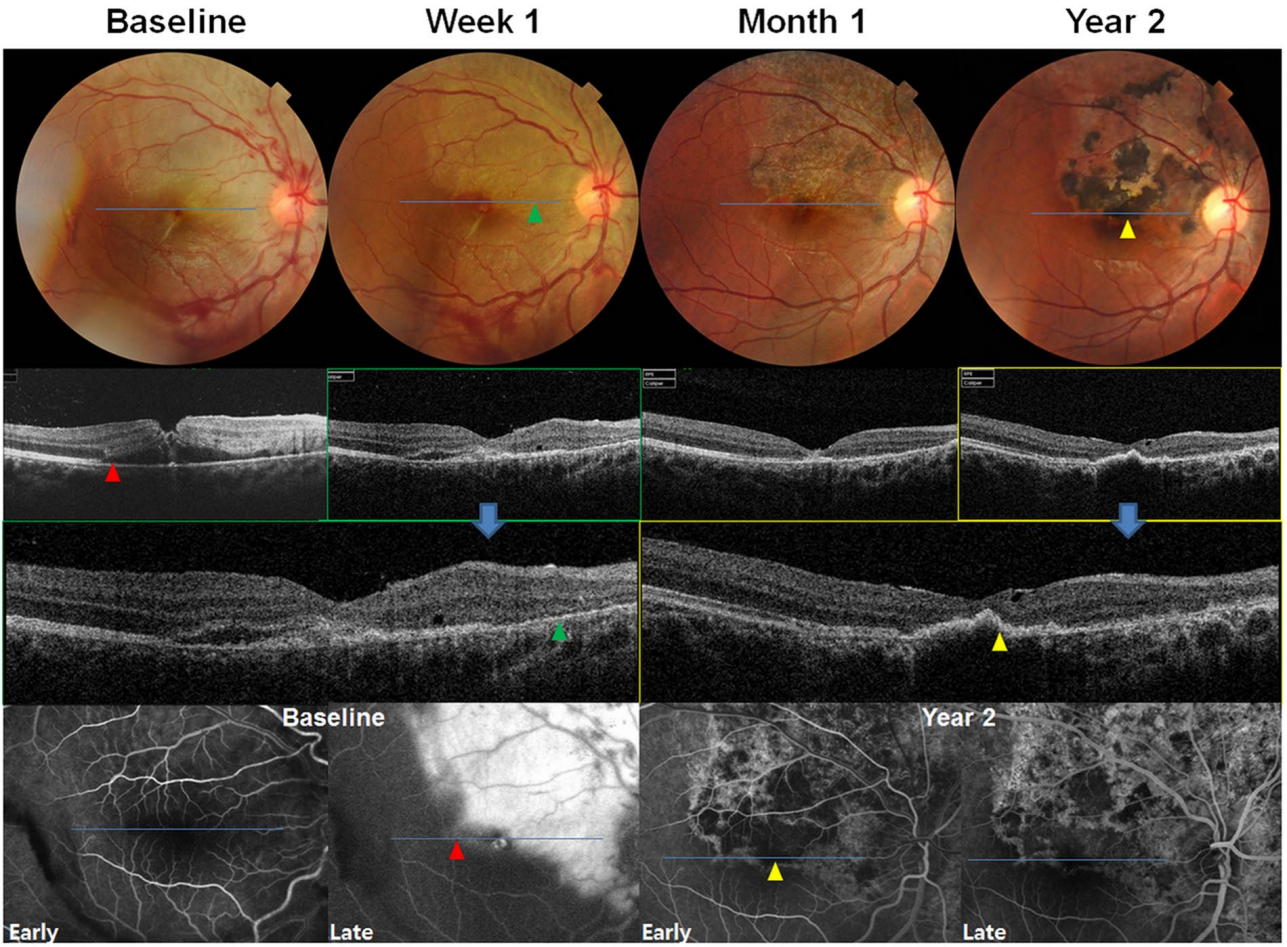
Fundus photographs (Top) and optical coherence tomography (OCT, middle) images showing microscopic structural changes in the eye with retinal pigment epithelium (RPE) sequelae. At baseline, a greyish or yellowish colour change is noted in the fundus photograph, and subretinal fluid (SRF) without definite vitreoretinal interface abnormality is noted on OCT. Fluorescein angiography (FA) images showing leakage and pooling at the late phase, which topographically corresponds to SRF (red arrowheads). At week 1, as SRF resolved in the nasal area to the fovea, a well-demarcated hypopigmentation (upper green arrowhead) corresponding to thinning of the RPE/Bruch’s membrane layer (lower green arrowhead) is noted in the OCT images. After 1 month, hyperpigmentation occurred within the hypopigmented lesion, which became prominent at year 2. Hyperpigmentation corresponds to a hyperreflective and thick RPE line (yellow arrowheads). FA images acquired at that time showing hypofluorescence on the hyperpigmentation and hyperfluorescence of the hypopigmented area, owing to blocked fluorescence and window defect, respectively. In both hypopigmented and hyperpigmented lesions, complete photoreceptor loss is commonly observed in the OCT images.

Figure 1 demonstrates a photographic example of RPE sequelae that developed in an eye with blunt ocular trauma. Figure 1A shows well-demarcated hypopigmentation and hyperpigmentation, which were observed at the final visit and which corresponded to an inferior visual field defect (A-1). Figure 1B shows a well-demarcated hypopigmented lesion with numerous hyperpigmented dots. The area showing RPE sequelae showed corresponding defects on visual field examination (B-1) and decreased amplitude on multifocal electroretinography (B-2). Overall, RPE sequelae were observed in 29 of 129 (22.5%) eyes at the final visit. At the 1-month follow-up, the incidence of RPE sequelae was 22.5% (29 of 129), and thus, no additional cases of development of RPE sequelae were noted after the first month (Fig. 1C). While RPE sequelae first occurred within 1 month in our patients, the area or pigmentation of such sequelae may progress over the follow-up period (Figs 2 and 3). Foveal involvement was observed in 14 patients, corresponding to 10.9% of all patients and 48.3% of patients showing RPE sequelae. Among the RPE sequelae observed, well-demarcated hypopigmentation was observed in all these patients, while hyperpigmented dots or areas within the hypopigmented lesion were observed in 25 of 29 eyes (86.2%). The location of RPE sequelae was mostly in the superior retina (24 of 29, 82.8%), symmetric around the macula (1 of 29, 3.4%), and inferior retina (4 of 29, 13.8%). Typically, the shape of the RPE sequelae resembled a comet, radiating from the optic disc (19 of 29 [65.5%], as shown in Fig. 1B). Others (10 [34.5%], as shown in Fig. 1) showed amorphous features without any connection to the disc. The appearance of hypopigmentation usually preceded that of the hyperpigmented lesion; the mean time from blunt trauma to the detection of hypopigmentation was 1.6 ± 0.76 weeks (range: 0–4 weeks) and that of hyperpigmentation was 2.9 ± 1.1 weeks (range: 1–6 weeks).

### Funduscopic and OCT features of RPE sequelae over time

Figure 2A demonstrates the development of RPE sequelae and their change over the 5 years following blunt ocular trauma. At baseline (Fig. 2A, top), commotio retinae over the retina superior to the fovea was noted, and FA showed a stippled area of hypofluorescence and mild hyperfluorescence at the early phase and leakage over the area at the late phase. Subsequently, the area showed hyperpigmentation within a hypopigmented lesion and the hyperpigmentation showed gradual enlargement over 5 years (Fig. 2A, bottom). Figure 2B indicates the gradual increase in the area of hyperpigmentation over time, represented by the mean area of hyperpigmentation, in eyes showing hyperpigmentation.

Figure 3 shows the microscopic outer retinal changes in the eye that developed RPE sequelae following blunt ocular trauma. At baseline, commotio retinae, linear choroidal rupture across the fovea, and retinal haemorrhage were noted in the fundus photographs (top) and OCT images (two middle frames) indicated the presence of SRF. Seven-line raster scan images (Supplementary Figure 1) covering the foveal and perifoveal areas revealed no vitreoretinal interface abnormality or macular hole. FA images (bottom) at baseline show leakage and pooling at the late phase, and the area corresponded to that of the SRF on OCT (red arrowheads). More specifically, the increase in intensity and area of hyperfluorescence over time in four FA images (Supplementary Figure 2) acquired from 13 to 334 s after intravenous fluorescein injection indicated the presence of leakage. Supplementary Figure 3 also shows fundus photographs and FA images from four other representative patients at baseline, which also reveal fluorescein leakage in patients with forthcoming RPE sequelae. Subsequently, at week 1, the retina superior or nasal to the fovea showed relatively well-demarcated hypopigmented areas (Fig. 3). The hypopigmented area (green arrowheads) showed thinning of the line of RPE/Bruch’s membrane complex in the OCT images, which may be attributed to bare Bruch’s membrane caused by the loss of the RPE. The OCT images showed complete loss of the photoreceptor layers over the defective RPE/Bruch’s membrane complex. Hyperpigmented dots (yellow arrowheads) within the hypopigmented area were noted after 1 month, which corresponded to RPE elevation in the OCT images. At year 2, the hyperpigmented area enlarged, presenting as a more elevated, thickened, and hyperreflective RPE layer in the OCT image. The image also revealed retinal thinning with remarkable photoreceptor defects. FA images acquired at that time showed hypofluorescence on the hyperpigmented area (due to blocked fluorescence) and hyperfluorescence on the hypopigmented area from the early phase (due to a window defect). The BCVA was not improved, and was 20/200 at both baseline and the final visit. In contrast, Supplementary Figure 4 demonstrates the case without RPE sequelae, in which the BCVA significantly improved from 20/200 at baseline to 20/25 at month 6 as the photoreceptor layers recovered.

After the first appearance of RPE sequelae, these changes persisted without any improvement in all patients. In 23 patients with RPE sequelae (79.3%), the area of hyperpigmentation increased over time, as shown in Fig. 2, while the others (20.7%) showed no progression in the size. As shown in Fig. 3 and Supplementary Figure 5, complete photoreceptor recovery was not obtained in any patient with RPE sequelae during the follow-up period.

### Visual function in eyes with RPE sequelae

**Figure 4:**
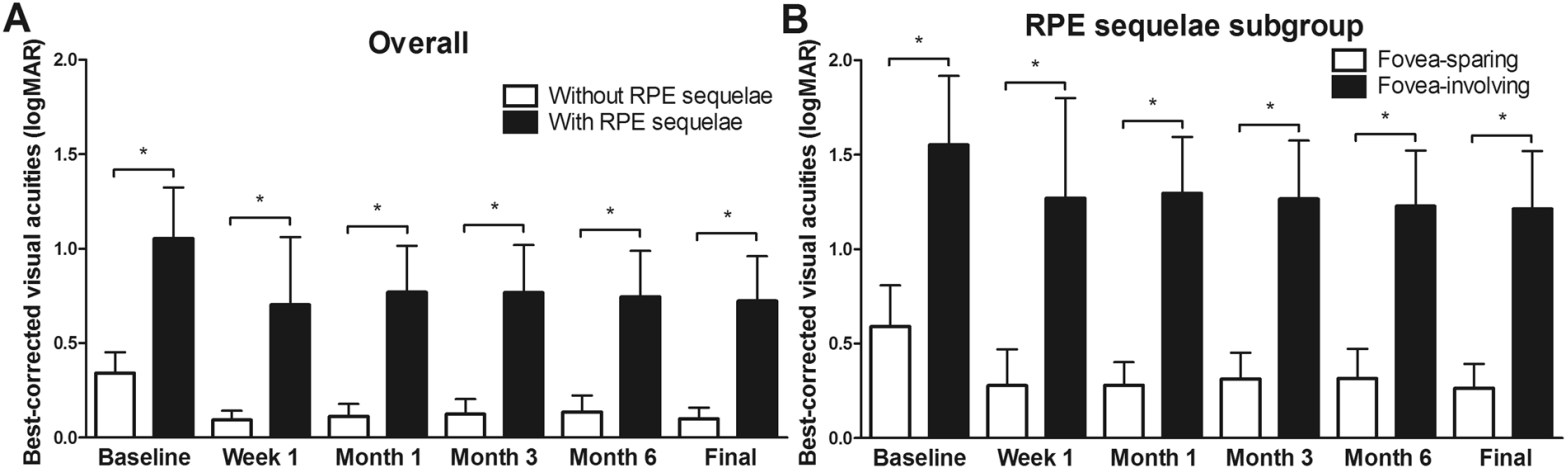
(**A**) Best-corrected visual acuities (BCVAs) over time in eyes with and without retinal pigment epithelium (RPE) sequelae and (**B**) those in eyes with fovea-sparing (non-foveal) and fovea-involving (foveal) RPE sequelae. At all baseline and follow-up visits, significant differences are noted in visual function between eyes with and without RPE sequelae, and between eyes with and without foveal involvement of RPE sequelae. Asterisk (*) indicates statistical significance. Error bars indicate upper bounds of 95% confidence intervals.

Figure 4 illustrates the visual function in eyes with and without RPE sequelae. Mean BCVA at baseline was 1.55 ± 0.63, 0.59 ± 0.39, and 0.34 ± 0.55 logMAR in eyes with fovea-involving (foveal) RPE sequelae, fovea-sparing (non-foveal) RPE sequelae, and without RPE sequelae, respectively.

At the final visit, the mean BCVA was 1.21 ± 0.53, 0.26 ± 0.23, and 0.10 ± 0.30 logMAR in eyes with foveal RPE sequelae, non-foveal RPE sequelae, and without the sequelae, respectively. In contrast to the eyes with non-foveal RPE sequelae or those without RPE sequelae, which showed significant improvement in mean BCVA from baseline to the final visit, eyes with foveal RPE sequelae showed no significant change between the baseline and final BCVA (*P* = 0.310). Furthermore, a significant difference was observed in the final BCVA between eyes with and without RPE sequelae (*P* < 0.001 by Student’s *t*-test). Among eyes with RPE sequelae, a significant difference was observed in the BCVA between those with and without foveal involvement (*P* < 0.001 by Mann-Whitney test).

### Comparison of clinical and ocular characteristics between eyes with and without RPE sequelae

Clinical characteristics, funduscopic features, and OCT findings at baseline were compared between eyes with and without RPE sequelae (Table 1). In addition to baseline BCVA, eyes with RPE sequelae had significantly more frequent choroidal rupture and SRF at baseline (*P* < 0.001) than did eyes without RPE sequelae. The number of eyes with commotio grades 0, 1, 2, 3, and 4 were 0, 0, 0, 15, and 11, respectively, in the group ‘With RPE sequelae’ and 11, 64, 11, 4, and 3, respectively, in the group ‘Without RPE sequelae’, showing a significant difference between the groups (P < 0.001 by Fisher’s exact test).

Logistic regression using several clinical factors showing borderline or statistical significance from the comparison between eyes with and those without RPE sequelae, including refractive errors, baseline BCVA, orbital wall fracture, SRF on baseline OCT, choroidal rupture, and commotio retinae grade, was also performed to determine factors associated with the development of RPE sequelae. From this analysis, SRF on baseline OCT was significantly associated with RPE sequelae (*P* = 0.004), with an odds ratio of 37.9 (95% confidence interval: 3.11–463.5).

## Discussion

This study showed that approximately 20% of patients with blunt ocular trauma and associated retinal abnormalities may have RPE sequelae. Evaluation of RPE sequelae is clinically important because they may be associated with persistent photoreceptor defects. Functionally, these can be associated with permanent visual loss or visual field defects, depending on the location of the RPE sequelae.

The functions of the RPE include secretion of essential factors and metabolic support for photoreceptor health. Thus, interactions between the RPE and photoreceptor cells are essential for the structural and functional integrity of the photoreceptors. Previous studies on some hereditary retinal degeneration have revealed a strong interdependence between the photoreceptors and RPE^9–14^. Furthermore, histological evidence suggests that RPE dysfunction plays a crucial role in the development of age-related macular degeneration, characterised by progressive loss of macular photoreceptors and central vision^15,16^. On the basis of the findings of previous studies, the interaction between the RPE and photoreceptors might explain persistent photoreceptor loss and visual decline in eyes with RPE sequelae following trauma. Alternatively, on the basis of the symbiotic relationship between the RPE and photoreceptors, the photoreceptor damage caused by blunt trauma may lead to RPE sequelae, which have been shown in previous experimental studies^17,18^. Our results on the association between the RPE and photoreceptor damage cannot help draw a conclusion on which occurs first, and clarifying this will require further longitudinal, histopathologic studies.

Our findings of RPE sequelae are consistent with the findings of ‘RPE oedema’ described by Friberg *et al*.^7^; the authors reported a cream-coloured discolouration of the RPE and subtle pigment clumping following ocular trauma. However, fundus and OCT images obtained from our patients suggest that the discolouration may arise from multiple causes including commotio retinae. While the photoreceptors usually recover over time in macular commotio retinae without RPE sequelae^6,19^, the eyes with RPE sequelae showed persistent visual loss and structural (photoreceptor) defects. Therefore, clinically, the development of RPE sequelae may be more important for understanding and predicting visual outcomes and photoreceptor recovery than are simple colour changes of the fundus at baseline, described as RPE oedema or RPE contusion.

We observed that one factor associated with RPE sequelae was the presence of SRF. For eyes with SRF, we confirmed the absence of a retinal hole or vitreoretinal interface abnormality (i.e. vitreoretinal traction) associated with SRF using multiple OCT images. The presence of SRF may indicate barrier function failure of the RPE^20^, caused by mechanical damage to the RPE. Alternatively, fluid at the subretinal level itself, regardless of the origin, may result in RPE sequelae or atrophy after its resolution, as has been demonstrated in several diseases such as central serous chorioretinopathy and retinal detachment^21^. Within the retinal area with SRF, dye leakage associated with defective barrier function was confirmed by FA, as shown in Figs 2 and 3. Interestingly, areas of fluorescein leakage corresponded well to subsequently developed RPE sequelae. Thus, the findings obtained from FA suggest that direct damage to the RPE leads to SRF at the acute phase and subsequent RPE sequelae. In this regard, colour changes of the fundus with SRF on OCT and dye leakage on FA can be referred to as a ‘RPE contusion’, which requires particular attention because of the likelihood of future development of RPE sequelae. However, it is also possible that the area with fluorescein leakage may be the area with severe commotio retinae, accompanied by severe photoreceptor damage; moreover, on the basis of the close relationship between photoreceptors and RPE damage, RPE sequelae may develop in this area.

The close relationship observed between RPE sequelae and photoreceptor defects suggests that RPE sequelae could be used clinically as an indicator of present and future photoreceptor loss. Whether RPE sequelae directly contribute to photoreceptor loss or photoreceptor damages precede RPE sequelae remains uncertain; however, our results on the association between the two suggest that the clinical evaluation of RPE sequelae may provide useful information on anatomical (photoreceptor status) and visual outcomes. Even without the use of a modern imaging modality such as OCT, a fundus examination or photograph can easily help identify the presence of RPE sequelae, from which the photoreceptor status of the area can be inferred.

In addition to the clinical evaluation of RPE sequelae by fundus examination or photography, our study suggests that eyes with retinal abnormalities caused by blunt trauma may require OCT at baseline. OCT is very useful as it indicates the presence of SRF at baseline. In traumatised eyes with hyphaema or vitreous haemorrhage, the media is not clear and a fundus examination or photograph may not be sufficient to determine the presence of SRF. Furthermore, in eyes with macular commotio retinae, the colour change associated with commotio retinae mimics that of SRF, which makes commotio retinae eyes with and without SRF indistinguishable. In such cases, OCT evaluation may help elucidate the presence of SRF, facilitating the prediction of RPE sequelae and photoreceptor recovery. Additionally, baseline FA may be useful to predict the area of RPE sequelae, particularly the foveal involvement of RPE sequelae, which might greatly affect the final visual outcome. However, on the basis of our results obtained from a small number of patients in whom FA was performed, we cannot draw a conclusion on whether FA is required for patients with blunt ocular trauma. Furthermore, FA, an invasive imaging modality, has potentially serious adverse effects such as bronchospasm and anaphylactic shock; therefore, it should be carefully performed, especially in patients in whom the benefits may outweigh the possible adverse effects.

Our study has several limitations that require careful interpretation. Owing to its retrospective design, it has the intrinsic drawback of a possible selection bias. In addition, the variable follow-up periods among our patients limited the results, particularly on final visual outcomes. Prospective studies with longer, uniform follow-up periods are needed to draw more definitive conclusions. Furthermore, our study cannot exclude the possibility of RPE sequelae occurring in the retinal periphery, which cannot be covered by conventional 45° fundus photography. The three hospitals included in this study were tertiary referral hospitals which patients with severe blunt trauma were more likely to visit; thus, the incidence of RPE sequelae among the eyes with retinal abnormalities might be higher than that in other populations. As RPE sequelae usually develop several weeks after traumatic events, it may not be used as an indicator of poor visual outcome at baseline. Thus, although its presence may help understand the cause of visual loss in eyes with poor visual outcome following blunt trauma, it cannot be clinically used for the prediction of anatomical and visual outcomes or treatment at baseline.

In conclusion, we observed that approximately 20% of patients with retinal abnormalities caused by blunt ocular trauma had RPE sequelae, usually hyperpigmentation within a well-demarcated hypopigmented lesion. This may be associated with permanent photoreceptor loss, and thus, depending on the location of RPE sequelae, may lead to significant visual loss or visual field defects. Therefore, during the clinical evaluation of eyes with retinal abnormalities caused by blunt trauma, particular attention must be paid to the presence of RPE sequelae. In particular, OCT may be clinically useful for the prediction of RPE sequelae.

## Methods

### Subjects

This study was based on a cohort of patients with blunt ocular trauma treated at the Armed Forces Capital Hospital, Seoul National University Bundang Hospital, and Hanyang University Hospital between January 2009 and January 2016. In total, 2289 patients with blunt ocular trauma were identified by searching the clinical database or electronic medical records of the three hospitals for the 10th revision of the International Statistical Classification of Diseases and Related Health Problems diagnostic codes: contusion, S001 or S051; other injuries of the eye and orbit, S058; other retinal disorders, H358 or H359; and free-text entries: commotio, trauma, injury, or assault. Clinical records were examined to include patients who were determined to have retinal abnormalities associated with blunt ocular trauma, confirmed by ophthalmologists on the basis of the following criteria: (1) history of acute blunt ocular trauma (within 7 days), and (2) unilateral retinal abnormalities in the traumatised eye. Patients who did not undergo standard fundus photography at baseline were excluded, and a patient cohort with 152 patients was established. Furthermore, patients were excluded if they had a follow-up period ≤ 1 month (n = 17), history of previous trauma or intraocular surgery, amblyopia, media opacity or macular/retinal diseases affecting the visual acuities before the trauma event, retinal detachment requiring vitreoretinal surgery (n = 3), or combined traumatic optic neuropathy (n = 1). We additionally excluded patients with choroidal rupture and associated linear pigmentary change, either hyperpigmentation or hypopigmentation (n = 2), as the RPE sequela is a presentation of choroidal rupture. After exclusion, 129 eyes with retinal abnormalities, identified by fundus photographs and caused by acute blunt trauma, were included in our analyses (Supplementary Figure 6). This study was approved by the Institutional Review Board of each hospital, and the study was conducted according to the tenets of the Declaration of Helsinki.

### Evaluation

Clinical records were examined to collect clinical information on demographic features, injury mechanism, and clinical findings on ophthalmologic examinations. Ophthalmologic examinations included best-corrected visual acuity (BCVA) measurements using Snellen charts, slit-lamp examinations, intraocular pressure measurements using noncontact tonometers (KT-500 automated tonometer; Kowa Co. Ltd., Tokyo, Japan), and indirect ophthalmoscopy. OCT examinations were performed using SD-OCT devices (Spectralis OCT [Heidelberg Engineering, Heidelberg, Germany] at Seoul National University Bundang Hospital, Cirrus OCT [Carl Zeiss Meditec, Dublin, CA] at Armed Forces Capital Hospital, and 3D-OCT 2000 [Topcon Inc., Tokyo, Japan] at Hanyang University Hospital); two line scans (6 mm in length), horizontal and vertical, across the fovea were used for our analyses. Fundus photographs, acquired using a mydriatic 45° fundus camera, either VX-10α (Kowa Inc., Nagoya, Japan) or a high-resolution fundus camera embedded in 3D-OCT 2000, were reviewed to identify the presence of retinal abnormalities at baseline and subsequent development of RPE sequelae. The results from a visual field examination (program 30–2 Swedish interactive threshold algorithm standard, model 750; Carl Zeiss Meditec, Dublin, CA) and those from multifocal electroretinography (VERIS II; Electro-Diagnostic Imaging, Inc., Redwood City, CA), if available, were reviewed. Fluorescein angiography (FA) images were also reviewed. FA was performed mostly in patients with subretinal fluid (SRF) in order to identify the source of fluid or the presence of leakage. Overall, FA was performed in 15 patients, including 13 and 2 with and without SRF at baseline, respectively.

A previously reported grading scheme based on photoreceptor status was used to document the severity of macular commotio retinae at baseline^6^. In brief, eyes with macular commotio retinae were graded on the basis of the photoreceptor status on SD-OCT as follows: Grade 1, an increase in reflectivity of the ellipsoid zone (EZ) with the disappearance of the thin hyporeflective optical space between the photoreceptor layers; Grade 2, an interdigitation zone (IZ) defect only; Grade 3, EZ and IZ defects; and Grade 4, IZ, EZ, and external limiting membrane (ELM) defects. For image analyses, we confirmed the locations and positions of the scans by using simultaneous fundus photographs or confocal scanning laser ophthalmoscope infrared reflectance images of the retina; this was done to ensure that follow-up scans were performed over the same macular area. Complete photoreceptor loss was defined as indistinguishable photoreceptor layers, namely, the IZ, EZ, and ELM. Conversely, complete photoreceptor recovery was defined as the presence of three structurally intact photoreceptor layers, with no defects in the layers^6^.

Calculation of the area of hyperpigmentation was performed as described in previous reports^22^. In brief, we manually merged the shadowgram images of a 6 × 6-mm area obtained by OCT and fundus photography using the vessel pattern by employing Photoshop Elements software (Adobe Systems, Inc., San Jose, CA, USA). The area of hyperpigmentation was measured using ImageJ (National Institutes of Health, Bethesda, MD, USA). The areas, in pixels, were converted to square millimetres.

As no definitive beneficial treatment for macular commotio retinae caused by acute blunt trauma has been reported, no patient received any specific treatment for macular commotio retinae. One patient with a massive subretinal haemorrhage (over half of the posterior pole) was treated using an intravitreal injection of sulphur hexafluoride followed by face-down positioning. For the treatment of hyphaema, patients received topical steroids and cycloplegics. Eyes with peripheral retinal tear and focal retinal detachment were treated with barrier laser photocoagulation.

### Analyses

Descriptive statistics were used for patient demographics, causes of trauma, and ocular characteristics such as refractive errors and axial lengths, anterior and posterior segment findings, and coexistence of orbital wall fracture. Student’s *t*-tests were used to compare the clinical characteristics and visual acuities between eyes with and without RPE sequelae. The Mann-Whitney test was used to compare eyes with fovea-sparing RPE sequelae and those with fovea-involving changes.

Logistic regression analyses were performed to identify the clinical factors or imaging findings significantly associated with the development of RPE sequelae. Continuous values are presented as the means ± standard deviation. P-values less than 0.05 were considered statistically significant. Statistical analyses were performed using SPSS Version 18.0 (SPSS Inc., Chicago, IL).

## Electronic supplementary material

Supplementary Information
