# Supplementary material for: Retinal Pigment Epithelium Sequelae Caused by Blunt Ocular Trauma: Incidence, Visual Outcome, and Associated Factors
Source: Sci Rep. 2017 Oct 27;7:14184. doi: 10.1038/s41598-017-14659-4 (PMC5660211; doi:10.1038/s41598-017-14659-4)
Supplement: Supplementary file 1 — Supplementary Information [file 41598_2017_14659_MOESM1_ESM.pdf]

Supplementary Information

Retinal Pigment Epithelium Sequelae Caused by Blunt Ocular Trauma: Incidence, Visual Outcome, and Associated Factors

Seong Joon Ahn, Se Joon Woo, Kyu Hyung Park, Byung Ro Lee

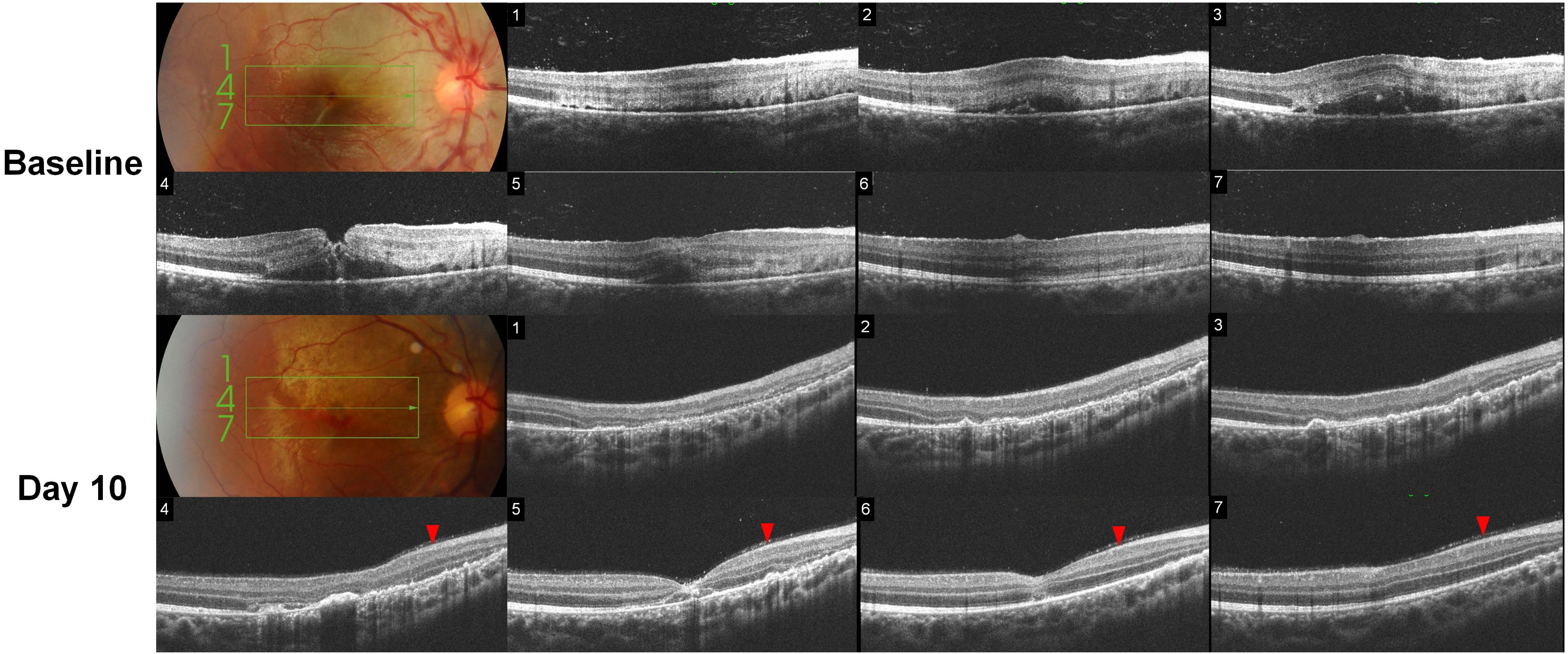

Supplementary Figure 1. Fundus photographs (top left) and optical coherence tomography (OCT) images showing the vitreoretinal interface on the foveal and perifoveal area in the eye with retinal pigment epithelium (RPE) sequelae. Red arrowheads indicate the posterior hyaloid membrane. At baseline and Day 10, no definite vitreoretinal interface abnormality is noted.

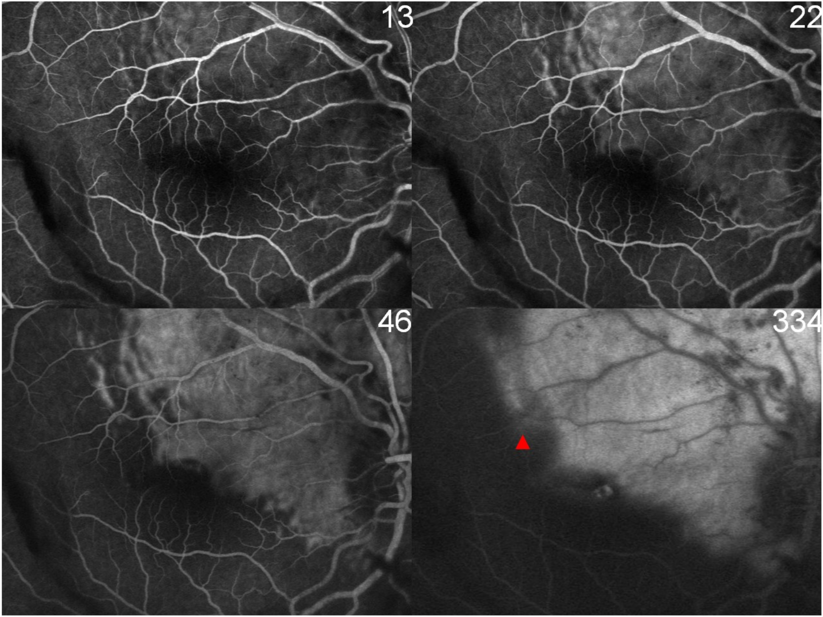

Supplementary Figure 2. Four fluorescein angiographs (FA) obtained in the eye with retinal pigment epithelium (RPE) sequelae. Text (upper right corner) indicates the time (seconds) at which FA was taken. Red arrowhead at the late phase image indicates the presence of leakage.

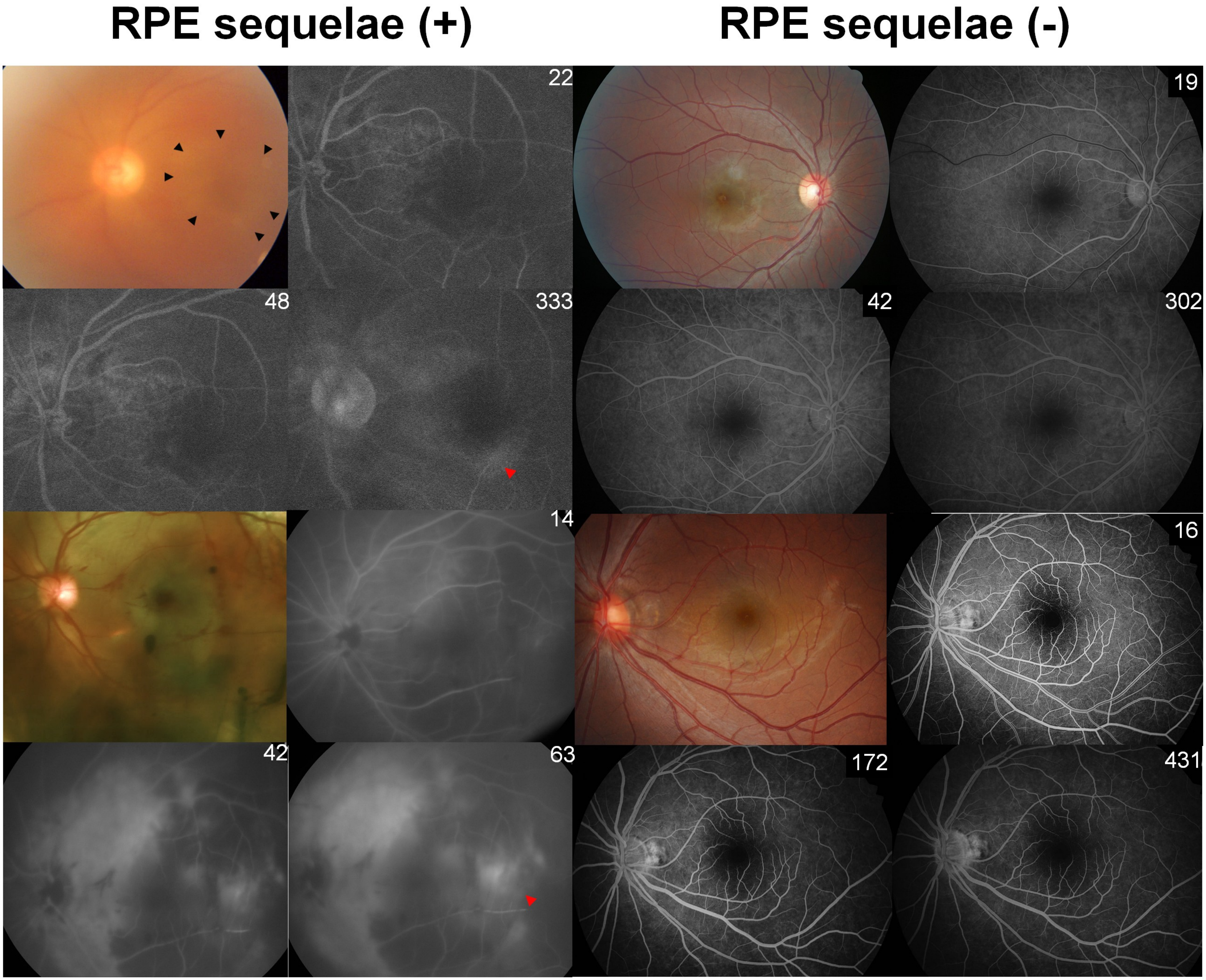

Supplementary Figure 3. Baseline fundus photographs (top left) and fluorescein angiographs (FA) in eyes with and without retinal pigment epithelium (RPE) sequelae. Whereas those with RPE sequelae show leakage at the late-phase FA images, those without RPE sequelae show no abnormal findings on FA. Texts on the upper-right corners denote the time (seconds) at which FA were taken.

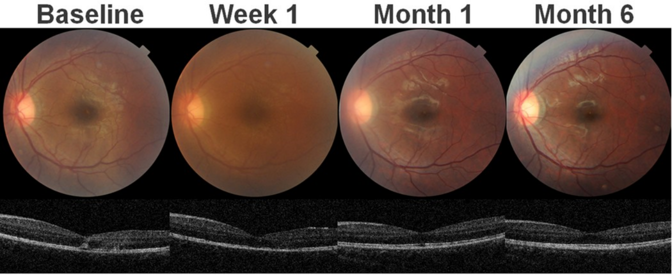

Supplementary Figure 4. Fundus photographs (upper) and optical coherence tomography (OCT, lower) images in the eye without retinal pigment epithelium (RPE) sequelae. At baseline, macular commotio retinae in fundus photograph and photoreceptor defects on OCT were noted. Subsequently, the photoreceptor layers completely recovered and no definite fundus abnormalities were noted at the final visit, month 6.

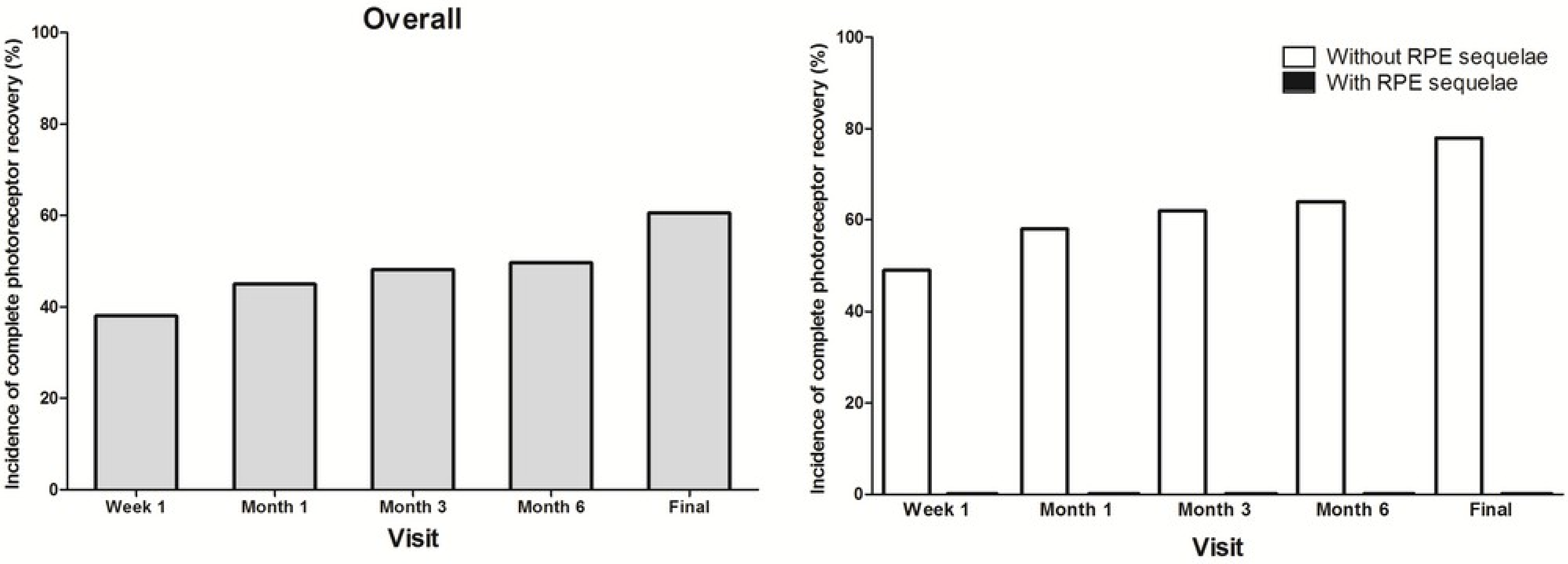

Supplementary Figure 5. Incidence of complete photoreceptor recovery in overall patients (left) and in those with and without RPE sequelae (right) over time.

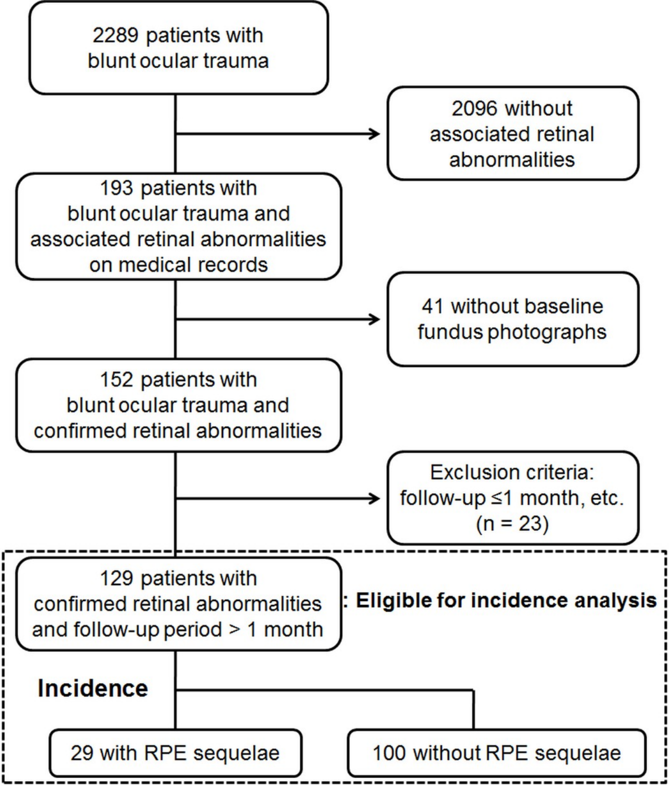

Supplementary Figure 6. Flowchart showing the study participants.

Supplementary Table. Causes of blunt ocular injury

Category or cause of trauma	Number (%) of patients
**Sports or leisure-related injury**	**81 (62.8)**
Soccer ball	59 (45.7)
Baseball	13 (10.1)
Foot	2 (1.6)
Tennis ball	2 (1.6)
Baseball bat	1 (0.8)
Basketball	1 (0.8)
Golf ball	1 (0.8)
Snowball	1 (0.8)
Rugby ball	1 (0.8)
**Work or training-related injury**	**19 (14.7)**
Paintball (guns)	4 (3.1)
Plastic	2 (1.6)
Rubber or rubber band	2 (1.6)
Wood	2 (1.6)
Stone	2 (1.6)
Iron plate	2 (1.6)
Spring	2 (1.6)
Nail	1 (0.8)
Antenna	1 (0.8)
Gun hinge	1 (0.8)
**Household injury**	**5 (3.9)**
Glasses	2 (1.6)
Refrigerator	1 (0.8)
Chair	1 (0.8)
Ruler	1 (0.8)
**Violence**	**18 (14.0)**
Fist	12 (9.3)
Elbow	4 (3.1)
Foot	2 (1.6)
**Others**	**6 (4.7)**
Traffic accident	3 (2.3)
Fall	2 (1.6)
Telephone pole	1 (0.8)
